# Pain Progression at Initiation of Cabazitaxel in Metastatic Castration-Resistant Prostate Cancer (mCRPC): A Post Hoc Analysis of the PROSELICA Study

**DOI:** 10.3390/cancers13061284

**Published:** 2021-03-13

**Authors:** Nicolas Delanoy, Debbie Robbrecht, Mario Eisenberger, Oliver Sartor, Ronald de Wit, Florence Mercier, Christine Geffriaud-Ricouard, Johann de Bono, Stéphane Oudard

**Affiliations:** 1 Medical Oncology, Université de Paris, 75015 Paris, France; nicolas.delanoy@aphp.fr; 2 Medical Oncology, AP-HP Paris, Centre, Georges Pompidou European Hospital, 75015 Paris, France; 3 Medical Oncology, Erasmus University Medical Center, 3000 CA Rotterdam, The Netherlands; d.robbrecht@erasmusmc.nl (D.R.); r.dewit@erasmusmc.nl (R.d.W.); 4 Medical Oncology, The Sidney Kimmel Comprehensive Cancer Center, Johns Hopkins University, Baltimore, MD 21231, USA; eisenma@jhmi.edu; 5 Medicine and Urology, Tulane Cancer Center, New Orleans, LA 70112, USA; osartor@tulane.edu; 6 Stat Process, Boulevard de Sébastopol, 75003 Paris, France; florence.mercier@statprocess.com; 7 Sanofi, Europe Medical Oncology, 75008 Paris, France; christine.geffriaud-ricouard@sanofi.com; 8 The Institute of Cancer Research, London SM2 5NG, UK; johann.de-bono@icr.ac.uk; 9 Royal Marsden NHS Foundation Trust, London SW3 6JJ, UK

**Keywords:** cabazitaxel, chemotherapy, clinical progression, metastatic castration-resistant prostate cancer, pain, taxanes, type of progression

## Abstract

**Simple Summary:**

Despite the emergence of new therapies during the last decade, metastatic castration-resistant prostate cancer (mCRPC) remains fatal. Recent work showed that the timing of treatment initiation seems critical for patient outcomes. Thus, it is key to identify factors that can help in deciding when to start treatment. In the PROSELICA prospective international phase III trial (NCT01308580), mCRPC patients received cabazitaxel at two dose levels. We performed a retrospective analysis to determine what type of disease progression patients displayed at the cabazitaxel initiation and how this progression affected the patient’s clinical outcomes. Pain progression was associated with aggressive disease and shorter survival, compared to other progression types (rise in serum PSA levels and/or alterations observed on CT scan or bone scan). Systematic classification of patients enrolled in future phase III trials according to disease progression at treatment initiation may help further practitioners to determine the best timeline for treatment initiation.

**Abstract:**

Background: In the PROSELICA phase III trial (NCT01308580), cabazitaxel 20 mg/m^2^ (CABA20) was non-inferior to cabazitaxel 25 mg/m^2^ (CABA25) in mCRPC patients previously treated with docetaxel (DOC). The present post hoc analysis evaluates how the type of progression at randomization affected outcomes. Methods: Progression type at randomization was defined as follows: PSA progression only (PSA-p; no radiological progression (RADIO-p), no pain), RADIO-p (±PSA-p, no pain), or pain progression (PAIN-p, ±PSA-p, ±RADIO-p). Relationships between progression type and overall survival (OS), radiological progression-free survival (rPFS), and PSA response (confirmed PSA decrease ≥ 50%) were analyzed. Results: All randomized patients (*n* = 1200) had received prior DOC, and 25.7% had received prior abiraterone or enzalutamide. Progression type at randomization was evaluable in 1075 patients (PSA-p = 24.4%, RADIO-p = 20.8%, PAIN-p = 54.8%). Pain progression was associated with clinical and biological features of aggressive disease. Median OS from CABA initiation or date of mCRPC diagnosis, all arms combined, was shorter in the PAIN-p group than in the RADIO-p or the PSA-p groups (12.0 versus 16.8 and 18.4 months, respectively, *p* < 0.001). In multivariate analysis, all arms combined, PAIN-p was an independent predictor of poor OS (HR = 1.44, *p* < 0.001). PSA response, rPFS, and OS were numerically higher with CABA25 versus CABA20 in patients with PAIN-p. Conclusions: This post hoc analysis of the PROSELICA phase III study shows that pain progression at initiation of CABA in mCRPC patients previously treated with DOC is associated with a poor prognosis. Disease progression should be carefully monitored, even in the absence of PSA rise.

## 1. Introduction

Several therapies have demonstrated an overall survival (OS) benefit in metastatic castration-resistant prostate cancer (mCRPC), including novel androgen receptor targeted therapies (ARTA; abiraterone, enzalutamide) [1,2], taxanes (docetaxel (DOC) [3,4,5], cabazitaxel (CABA) [6,7]), poly-ADP ribose polymerase inhibitors (olaparib, rucaparib) [8], immunotherapy (sipuleucel-T) [9], and a bone-targeted radiopharmaceutical (radium-223) [10]. However, mCRPC remains fatal. The latest improvements in disease management consist mainly of the use of DOC and ARTA at earlier stages of the disease in metastatic castration-sensitive prostate cancer (mCSPC) [11,12,13,14,15,16], and the use of ARTAs in non-metastatic castration-resistant prostate cancer (nmCRPC) [17,18,19]. Therefore, the timing of treatment initiation seems to play a critical role. In a post hoc analysis of three randomized phase III studies in first-line mCRPC (TAX-327, VENICE, FIRSTANA), we recently reported that pain progression at chemotherapy initiation was associated with worse outcome [20]. These findings were supported by the large international CATS registry, suggesting that clinical progression at the initiation of a life-extending therapy was associated with a shorter OS, not only in first-line mCRPC, but also in second and third-line settings, whatever the therapy (DOC, CABA, or ARTA) [21]. Moreover, clinical progression seemed to be associated with a shorter duration of therapy with ARTA compared with taxanes.

The large phase III randomized study PROSELICA (NCT01308580) evaluated the non-inferiority of two doses of CABA, at the standard dose of 25 mg/m^2^ every 3 weeks (CABA25) and the lower dose of 20 mg/m^2^ every 3 weeks (CABA20) in mCRPC patients previously treated with DOC [22]. The present post hoc analysis of PROSELICA further assesses the prognostic value of the type of disease progression at CABA initiation in a post-DOC setting.

## 2. Patients and Methods

### 2.1. Population

PROSELICA was a phase III randomized study evaluating the non-inferiority of CABA25 and CABA20 every 3 weeks with daily prednisone in 1200 mCRPC patients previously treated with DOC. The primary endpoint was OS. Inclusion criteria, evaluation criteria, and results have been published [22]. Patients enrolled in the study were symptomatic or not and had disease progression defined by progression of measurable lesions (RECIST 1.1 criteria) or non-measurable lesions (Prostate Cancer Working Group (PCWG) 2 criteria [23]), or PSA progression (PCWG2 criteria). Pain was recorded by the patient on a daily basis using the present pain intensity (PPI) scale of MacGill–Melzack [24]. The score ranged from 0 to 5, with higher scores indicating greater pain. In addition, a daily analgesic score (AS) was calculated for 7 days before randomization, assigning a score of 4 for a standard dose of narcotic analgesics and a score of 1 for a standard dose of non-narcotic analgesics [22].

### 2.2. Data Collection

Prostate-specific antigen (PSA) was measured at baseline and every 3 weeks during therapy. Chest, abdomen, and pelvic CT-scan or MRI were performed at baseline and every 6 weeks during therapy and repeated after 4 weeks to confirm progression. Bone scan was performed at baseline and every 12 weeks during therapy. In this post hoc analysis, all randomized patients were classified into three groups according to the type of disease progression at randomization [22]: PSA progression only (PSA-p) was defined by rising serum levels of PSA on at least two consecutive measurements obtained at least one week apart with a value of at least 2 ng/mL, without radiological progression and without pain; radiological progression (RADIO-p) was defined by radiologic progression on CT scan or bone scan, with or without rising PSA and without pain; pain progression (PAIN-p) was defined by mean present pain intensity (PPI) ≥ 2 (McGill–Melzack questionnaire) and/or mean analgesic score (AS) ≥ 10 over the 7 days prior to randomization [24], with or without PSA rise, with or without radiological progression. Patients were excluded if the type of progression was not evaluable.

### 2.3. Statistical Analysis

Our primary objective was to explore the prognostic impact of the type of disease progression at initiation of CABA, all arms combined, on OS. To control for a lead time bias, OS was also calculated from the date of diagnosis of mCRPC (estimated by the date of start of subsequent anticancer therapy after the first androgen deprivation therapy (ADT)).

Univariate and multivariate Cox regression analyses with backward elimination (5% level) were performed, all arms combined, stratified for the region of treatment (Asia, Europe and Australia, US and Canada, others), Eastern Cooperative Oncology Group performance status (ECOG-PS) (0–1 vs. 2) and disease status (measurable or not). The following variables were tested: Gleason score at diagnosis; duration of initial ADT; prior therapy with curative intention (radical prostatectomy and/or prostate radiation therapy); age, pain status (PPI ≥ 2 or analgesic score ≥ 10), and metastatic sites as per Halabi classification [25] (lymph nodes only, bone ± lymph nodes, visceral ± bone ± lymph nodes) at baseline; PSA levels and PSA doubling time (DT); testosterone, hemoglobin (Hb), alkaline phosphatase (ALP), and lactate-dehydrogenase (LDH) levels; absolute neutrophil count; and neutrophil-to-lymphocyte ratio (NLR) at randomization. Age, duration of initial ADT, PSA, PSA DT, Hb, ALP, LDH, neutrophil count, and NLR were dichotomized according to their medians. Fisher’s exact test was used for all categorical variables, and the Kruskal–Wallis test for all continuous variables.

Secondary objectives were to evaluate the impact of the type of progression in the 2 treatment arms on the following parameters: OS from randomization and mCRPC diagnosis, confirmed PSA decrease ≥ 50% from baseline, radiological progression-free survival (rPFS), and type of first progression event during therapy (PSA-p, RADIO-p, or PAIN-p). rPFS was defined as the time from randomization to the first radiological progression event diagnosed according to RECIST 1.1 (for measurable lesions) or PWCG2 criteria (for bone lesions), or death from any cause.

## 3. Results

### 3.1. Population

**Figure 1:**
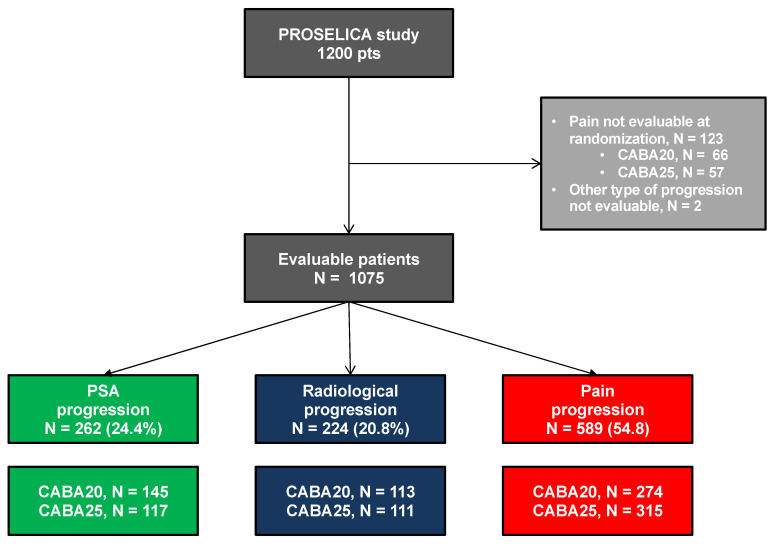
Flow chart. CABA20: cabazitaxel 20 mg/m^2^ every 3 weeks (Q3W), CABA25: cabazitaxel 25 mg/m^2^ Q3W, PSA progression: patient with a rising PSA only at randomization, Radiological progression: patient with a radiologic progression on CT scan or bone scan with (*N* = 177) or without (*N* = 47) PSA progression, Pain progression: patient with a mean present pain intensity (PPI) ≥ 2 (McGill–Melzack questionnaire) and/or mean analgesic score (AS) ≥ 10 over the 7 days prior to randomization with (*N* = 505) or without (*N* = 84) PSA progression or radiological progression.

A total of 1200 patients with mCRPC were enrolled between April 2011 and December 2013, of whom 598 and 602 were randomly assigned to receive CABA20 and CABA25, respectively (Figure 1). The type of disease progression at randomization was evaluable in 1075 patients (89.6%) (Figure 1). PAIN-p was the most common type of progression (54.8%), followed by PSA-p (24.4%) and RADIO-p (20.8%). Median follow-up was 13.5 months.

Baseline characteristics at randomization according to the type of progression are presented in Table 1. Bone metastases were observed in a majority of patients whatever the progression group, but with a higher number of patients in PAIN-p (96.43%) as compared to PSA-p and RADIO-p (92.75% and 85.27% respectively). As compared to patients with PSA-p only, those with PAIN-p also had clinical and biological features of aggressive disease: higher rate of ECOG-PS 2 (15.8 vs. 3.1%), visceral metastases (30.9 vs. 14.9%); lower median values of hemoglobin (11.6 vs. 12.2 g/dL); higher median values of PSA (192.3 vs. 141.7 ng/mL), NLR (3.7 vs. 2.7), absolute neutrophil count (4.9 vs. 4.3 10*9/L), ALP (214 vs. 138 UI/L) and LDH (360 vs. 294 UI/L). Patients with RADIO-p had intermediate values between PSA-p and PAIN-p groups.

### 3.2. Impact of Baseline Type of Progression

**Figure 2:**
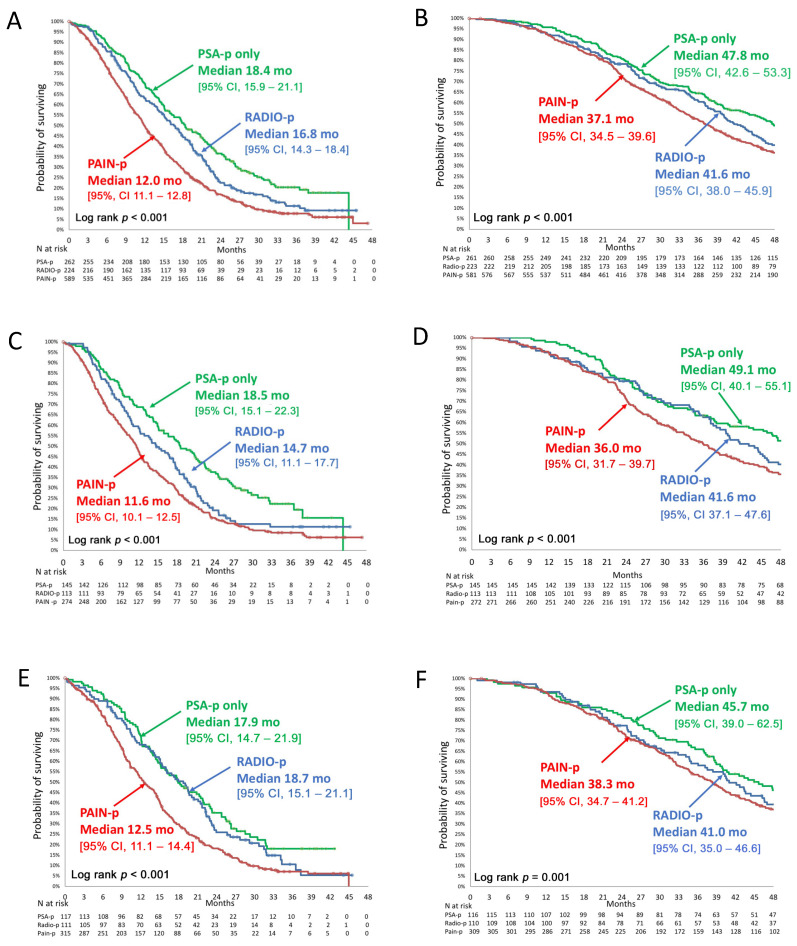
Overall survival according to the type of disease progression from date of cabazitaxel initiation and from date of mCRPC diagnosis. (**A**) Overall survival (OS) from cabazitaxel (CABA) initiation in ITT population all arms combined, (**B**) OS from date of mCRPC diagnosis all arms combined, (**C**) OS on CABA20 from CABA initiation, (**D**) OS on CABA20 from date of mCRPC diagnosis, (**E**) OS on CABA25 from CABA initiation, (**F**) OS on CABA25 from date of mCRPC diagnosis. CABA20: cabazitaxel 20 mg/m^2^ every 3 weeks (Q3W), CABA25: cabazitaxel 25 mg/m^2^ Q3W, mCRPC: metastatic castration-resistant prostate cancer, ITT: intention to treat population, PSA-p: PSA progression only, RADIO-p: radiological progression (with or without PSA-p), PAIN-p: pain progression (with or without PSA-p or RADIO-p).

Median OS from CABA initiation, all arms combined, was shorter in the PAIN-p group (Figure 2): 12.0 (95% CI, 11.1–12.8) months versus 16.8 (14.3–18.4) months in the RADIO-p group and 18.4 (15.9–21.1) months in the PSA-p group (*p* < 0.001). This effect was consistent in both treatment arms: median OS from CABA20 initiation was 11.6 (10.1–12.5) months in the PAIN-p group versus 14.7 (11.1–17.7) months in the RADIO-p group and 18.5 (15.1–22.3) months in the PSA-p group (*p* < 0.001); median OS from CABA25 initiation was 12.5 (11.1–14.4) months in PAIN-p versus 17.9 (14.7–21.9) months in the RADIO-p group and 18.7 (15.1–21.1) months in the PSA-p group (*p* < 0.001).

To avoid a lead time bias, median OS was also calculated from date of mCRPC diagnosis (Figure 2): median OS, all arms combined, was 37.1 (34.5–39.6) months in the PAIN-p group versus 41.6 (38.0–45.9) months in the RADIO-p group and 47.8 (42.6–53.3) months and in the PSA-p groups (*p* < 0.001). Similar findings were also observed in both treatment arms (Figure 2).

### 3.3. Multivariate Analysis

The multivariate analysis (Table 2) showed that the type of progression at CABA initiation was prognostic; PAIN-p was associated with a shorter OS compared to the reference group PSA-p (HR = 1.44, *p* < 0.001). Low hemoglobin (HR = 1.62), high ALP (HR = 1.47), high LDH (HR = 1.22), high neutrophil count (HR = 1.21), short PSA doubling time (HR = 1.3), high PSA levels (HR = 1.27), ECOG PS 2 (HR = 1.35), and presence of measurable disease (HR = 1.36) at baseline were also significantly associated with a worse OS (Table 2).

### 3.4. Impact of the Cabazitaxel Treatment Arm

Confirmed PSA response, all arms combined, was lower in the PAIN-p group than in RADIO-p and PSA-p groups (31.3% vs. 43.7% and 35.9%, respectively, *p* = 0.02) (Table 3). Analyzed by treatment arm, PSA responses were higher with CABA25 than with CABA 20, regardless of the type of progression.

The number of patients (all progression groups combined) who experienced a pain response (defined as a 2-point reduction in PPI score on the McGill–Melzack scale and/or a reduction of at least 50% of the analgesia score (AS)) was slightly higher in the CABA25 arm (37.3%) than in the CABA20 arm (34.7%), but this difference was not found significant (*p* = 0.4) (data not shown).

Median OS from CABA initiation was numerically higher but not statistically significant with CABA25 versus CABA20 in PAIN-p and RADIO-p groups: 12.5 (11.1–14.4) vs. 11.6 (10.1–12.5) months (*p* = 0.752) and 18.7 (15.1–21.1) vs. 14.7 (11.1–17.7) months (*p* = 0.109), respectively). In the PSA-p group, median OS was similar with CABA25 versus CABA20 (17.9 (14.7–21.9) versus 18.5 (15.1–22.3) months (*p* = 0.855), respectively). In the PSA-p group, OS was similar between CABA 25 (17.9 (14.7–21.9)) and CABA 20 (18.5 (15.1–22.3)).

Median rPFS, all arms combined, was also shorter in the PAIN-p group than in the RADIO-p and PSA-p groups (7.8 (6.9–8.4) vs. 8.1 (7.0–8.8) and 10.0 (9.3–11.3) months, respectively, *p* < 0.001) (Table 3). Analyzed by treatment arm, rPFS was numerically higher (but not statistically significant) with CABA25 than with CABA20 for patients with PAIN-p (8.2 versus 7.1 months, *p* = 0.653) and RADIO-p (8.7 versus 7.2 months, *p* = 0.098), but did not differ between arms for patients with PSA-p (9.8 versus 10.0 months with CABA25 vs. CABA20, respectively, *p* = 0.362).

Analysis of the type of progression order led to the identification of 16 patterns of progression (Table 4). PAIN-p was the first progression event during therapy in 39.4% (*n* = 424) of patients, followed by PSA-p only in 36.7% (*n* = 395) of patients, and RADIO-p in 12.5% (*n* = 134) of patients. PAIN-p without rising PSA was observed in 283 patients (26.3%), and RADIO-p without rising PSA was observed in 105 patients (9.8%) (Table 4).

## 4. Discussion

This post hoc analysis of the large, randomized phase III study PROSELICA confirms that PAIN-p at initiation of CABA in mCRPC patients previously treated with DOC is associated with a poor prognosis. First, patients with PAIN-p had clinical and biological features of aggressive disease, including higher rates of ECOG PS 2 and visceral metastases, lower hemoglobin values, and higher values of ALP, LDH, absolute neutrophil count, and NLR at treatment initiation as compared to PSA-p. Second, patients with PAIN-p at randomization had a worse OS versus those with PSA-p only, calculated from both CABA initiation (18.4 vs. 12.0 months, *p* < 0.001) and from mCRPC diagnosis (47.8 vs. 37.1 months, *p* < 0.001). Third, PAIN-p was an independent predictor of poor OS in multivariate analysis. Fourth, in patients with PAIN-p, CABA25 showed a numerically greater activity than CABA20 in terms of PSA response, rPFS, and OS. Lastly, PAIN-p was the most common first progression event during therapy (39.4%), followed by PSA-p only (36.7%) and RADIO-p (12.5%).

These data, obtained in a post-docetaxel setting, support the findings of Robbrecht et al. in a post hoc analysis of three phase III clinical trials (TAX327, VENICE, FIRSTANA) evaluating the efficacy of chemotherapy in first-line mCRPC [20]: pain at initiation of first-line chemotherapy was also associated with features of aggressive disease and was an independent prognostic factor of poor OS in multivariate analysis. More recently, a post hoc analysis of the COU-AA-302 study of abiraterone in chemo-naïve mCRPC patients [26] also showed that pain level at treatment initiation was prognostic, as well as ECOG-PS, PSA, LDH, and ALP levels at treatment initiation. An important difference between these studies was that COU-AA-302 included exclusively patients who were asymptomatic or with mild pain, while chemotherapy studies enrolled mainly patients with severe pain. Although phase III studies cannot be compared between each other, it is likely that pain severity at baseline contributed to the ~1-year difference in survival outcomes observed in chemotherapy studies in first-line mCRPC patients [3,22,27], and in phase III studies with abiraterone [28] and enzalutamide [2,29] in chemo-naïve patients. The poor prognostic value of clinical progression has also been observed at initiation of each therapy line in the large retrospective CATS registry [21,30] that enrolled 661 patients treated with 3 life-extending therapies (DOC, CABA, and one ARTA) in any order. Clinical progression defined by alteration of ECOG-PS or pain progression was the most common progression type, regardless the therapy line and its prevalence increased with the number of lines (from 43.1% at initiation of first-line therapy to 67.9% at initiation of third-line therapy). Clinical progression was consistently associated with clinical and biological features of aggressive disease and worse outcomes in terms of rPFS and OS, whatever the treatment line and treatment type (chemotherapy or ARTA).

Prostate cancer progression in phase III mCRPC trials is consistently defined by progression of measurable lesions (as per RECIST), or appearance of new bone lesions, or confirmed rising PSA (as per PCWG 2 or 3 criteria) [23,31]. We believe this definition of disease progression is insufficient because it does not take into account pain progression, which is a major prognostic factor consistently associated with features of aggressive disease in mCRPC as well as in mCSPC [32]. Moreover, there is increasing evidence that many patients do not follow the sequence of progression events model initially described [28]: “PSA progression” as the first progression event to occur during therapy, followed by “radiological progression”, followed by “clinical progression”. Indeed, in the PREVAIL study, one out of four patients had radiological progression without PSA progression [26]. In PROSELICA, pain progression was often the first progression event to occur, before any PSA or radiological progression, as previously reported in first-line chemotherapy studies (TAX327, VENICE, FIRSTANA) [20]. We thus propose to include pain progression in the definition of disease progression in future prostate cancer clinical studies. Methodologies used to measure pain progression should also be harmonized: chemotherapy phase III studies (TAX327, VENICE, FIRSTANA, PROSELICA) evaluated pain progression by means of the present pain intensity (PPI) scale from the McGill–Melzack questionnaire and an analgesic use diary [20], while abiraterone and enzalutamide phase III studies evaluated pain using the brief pain inventory short-form (BPI-SF) questionnaire and the WHO analgesic ladder [28,29]. This harmonization requires thorough review and collaborative work to achieve consensus on actions to be implemented.

The high frequency of bone metastases in all types of progression, and in particular in the PAIN-p group, highlights the unmet need for more frequent imaging evaluation. PSA is not the ideal biomarker since 25% of patients show clinical or radiological progression without any PSA rise. Therefore, bone imaging should be highly recommended as a regular evaluation in mCRPC patients [33].

Our data suggest that the type of disease progression may provide some guidance to tailor the CABA dosage of each patient. PROSELICA concluded that CABA20 is non-inferior to CABA25 in terms of OS, the primary end point, and had a favorable adverse event profile [22]. Based on these results, the starting dose of CABA has been reduced to 20 mg/m^2^ in the US but not in Europe. The recent phase III CARD study applied the 25 mg/m^2^ dose. The incidence of neutropenic fever was no more than 3.2%, possibly due to the use of systematic primary G-CSF prophylaxis [34]. Our analysis suggests that patients with pain progression may have a greater benefit from CABA25 on PSA response, rPFS, and OS. Similar findings have been observed in FIRSTANA in first-line mCRPC [27]. In patients with pain progression, median OS was higher in patients treated with CABA25 than those treated with CABA20 (20.4 versus 16.5 months, *p* = 0.0143) [27]. The slight difference observed in pain response between CABA25 and CABA20 does not suffice to justify the sole use of patient type of progression to determine the CABA dosage to be administered. We rather propose to take it as an additional parameter to take into account when making the decision. In any case, further investigation is required to support this proposal, such as a head-to-head comparison of patients with pain progression treated with different doses.

Our study has several limitations. Although we used a phase 3 clinical trial database, our analysis was not pre-planned and the classes of disease progression used were not pre-specified. However, pain at randomization was well documented and closely monitored by the mean of standardized and referenced scales over seven days before randomization, providing a robust evaluation of its intensity [22]. PSA values were measured every three weeks, CT-scan or MRI every six weeks, and bone scan every 12 weeks, providing a robust and a reliable evaluation of PSA and radiological progression. Another limitation is that PROSELICA enrolled highly selected patients, with well-controlled comorbidities and fit enough to receive chemotherapy, which may not reflect real-life practice. At the time PROSELICA was recruiting, abiraterone or enzalutamide were not available in all centers, explaining why only one out of four patients received them before cabazitaxel initiation. Nowadays, a vast majority of patients will receive ARTA before initiating chemotherapy [35,36].

## 5. Conclusions

This post hoc analysis of a large phase III study in mCRPC patients post-DOC further confirms that pain progression at CABA initiation is associated with clinical and biological features of aggressive disease and worse outcomes. Since pain progression and/or radiological progression may happen without rising PSA, it is crucial to carefully monitor mCRPC patients by performing regular imaging and symptom evaluation. Considering the strong impact of pain on outcomes, we suggest that patients enrolled in future phase III trials are to be systematically classified according to PSA, radiological, and pain progression at treatment initiation.

## Figures and Tables

**Table 1:** Patient characteristics at disease diagnosis and at cabazitaxel initiation by type of disease progression.

Characteristic	PSA-p *N* = 262	RADIO-p *N* = 224	PAIN-p *N* = 589	*p* Value
**Disease History**				
Gleason 8–10 at diagnosis (%)	49.2	48.1	55.4	0.106
Prior radical prostatectomy (%)	24.8	22.3	21.9	0.632
Prior radical radiotherapy (%)	27.1	37.1	28.0	0.019
M1 disease at diagnosis (%)	48.2	42.9	50.8	0.176
Median duration of response to first ADT (mths)	11.7	14.0	12.0	0.152
**Patients Characteristics at Cabazitaxel Initiation**				
Median age (years)	68	70	68	0.002
ECOG PS 2 (%)	3.1	1.3	15.8	<0.001
Metastatic sites by Halabi classes (%)				<0.001
Lymph nodes only	4.2	5.4	1.2	
Bone +/− nodes	71.4	46.9	55.9	
Visceral +/− bone or nodes	14.9	31.7	30.9	
Measurable lesions (%)	27.5	71.9	47.5	<0.001
Prior Abiraterone/Enzalutamide	21.8	23.7	28.4	0.092
Median PSA levels (ng/mL)	141.7	122.5	192.3	0.006
Median hemoglobin (g/dL)	12.2	12.4	11.6	<0.001
Median neutrophil-to-lymphocyte ratio	2.7	3.2	3.7	<0.001
Median neutrophil count (Giga/L)	4.3	4.4	4.9	<0.001
Median alkaline phosphatase (IU/L)	137.5	123.0	214.0	<0.001
Median lactate dehydrogenase (IU/L)	294.0	294.6	360.0	<0.001

*p*-values are global. PSA-p: PSA progression only, RADIO-p: radiological progression (with or without PSA-p), PAIN-p: pain progression with or without PSA-p or RADIO-p, ECOG PS: Eastern Cooperative Oncology Group performance score.

**Table 2:** Multivariate analysis of overall survival.

Characteristics at Baseline	Stratification	HR	95% CI	*p* Value
Alkaline phosphatase (median, 166 IU/L)	<median ≥median	Ref 1.47	[1.25–1.73]	<0.001
Hemoglobin (median, 12.0 g/dL)	≥median <median	Ref 1.62	[1.40–1.88]	<0.001
Lactate dehydrogenase (median, 327 IU/L)	<median ≥median	Ref 1.22	[1.05–1.42]	0.01
Type of progression	PSA-p RADIO-p PAIN-p	Ref 1.21 1.44	[0.96–1.51] [1.21–1.72]	<0.001
Neutrophil count (median, 4.7 g/L)	<median ≥median	Ref 1.21	[1.05–1.39]	0.009
PSA doubling time (median, 2 months)	≥median <median	Ref 1.3	[1.13–1.50]	<0.001
PSA level (median, 165.5 ng/mL)	<median ≥median	Ref 1.27	[1.09–1.47]	0.002
ECOG PS (Stratification factor)	0 or 1 2	Ref 1.35	[1.06–1.71]	0.01
Measurable disease (Stratification factor)	Non-measurable measurable	Ref 1.36	[1.17–1.58]	<0.001

Multivariate Cox regression analyses with backward elimination (5% level), stratified for the region of treatment (Asia, Europe and Australia, US and Canada, others), ECOG PS: Eastern Cooperative Oncology Group performance score (0–1 vs. 2), and disease status (measurable or not).

**Table 3:** PSA response, radiological-progression-free survival, and overall survival from randomization.

Treatment Arm	PSA-p *N* = 261	RADIO-p *N* = 223	PAIN-p *N* = 581	Global *p*
**PSA Response**				
OVERALL	35.9%	43.7%	31.3%	*p* = 0.02
CABA20	31.2%	33.7%	26.0%	*p* = 0.49
CABA25	41.8%	53.9%	36.0%	*p* = 0.02
**Radiological-Progression-Free Survival**				
OVERALL	10.0 [9.3; 11.3]	8.1 [7.0; 8.8]	7.8 [6.9; 8.4]	*p* < 0.001
CABA20	10.0 [9.0; 11.3]	7.2 [5.3; 8.3]	7.1 [6.0; 8.3]	*p* < 0.001
CABA25	9.8 [8.9; 14.7]	8.7 [7.2; 9.8]	8.2 [7.2; 8.9]	*p* < 0.001
**Overall Survival from Mcrpc Diagnosis**				
OVERALL	47.8 [42.6; 53.3]	41.6 [38.0; 45.9]	37.1 [34.5; 39.6]	*p* < 0.001
CABA20	49.1 [40.1; 55.1]	41.6 [37.1; 47.6]	36.0 [31.7; 39.7]	*p* < 0.001
CABA25	45.7 [39.0; 62.5]	41.0 [35.0; 46.6]	38.3 [34.7; 41.2]	*p* = 000.1
**Overall Survival from Randomization**				
OVERALL	18.4 [15.9; 21.1]	16.8 [14.3; 18.4]	12.0 [11.1; 12.8]	*p* < 0.001
CABA20	18.5 [15.1; 22.3]	14.7 [11.1; 17.7]	11.6 [10.1; 12.5]	*p* < 0.001
CABA25	17.9 [14.7; 21.9]	18.7 [15.1; 21.1]	12.5 [11.1; 14.4]	*p* < 0.001

PSA response defined as a decrease of PSA from baseline ≥ 50% on two subsequent PSA dosages according to the type of progression in overall population of PROSELICA (all arms combined), in CABA20 and in CABA25. Radiological Progression free survival, defined as the time from randomization to the first event occurring among radiological progression according to RECIST 1.1 or PWCG2 criteria or death from any cause, CABA20: Cabazitaxel 20 mg/m^2^ every 3 weeks (Q3W), CABA25: Cabazitaxel 25 mg/m^2^ Q3W.

**Table 4:** Patterns of progression by treatment arm and overall population.

Type of Progression	CABA20 *N* = 532	CABA25 *N* = 543	ALL *N* = 1075
PAIN first, *n* (%)	204	220	424 (39.4)
PAIN only	77	94	171 (15.9)
PAIN > PSA	32	41	73 (6.8)
PAIN > PSA >RADIO	43	39	82 (7.6)
PAIN > RADIO	32	33	65 (6.0)
PAIN > RADIO > PSA	20	13	33 (3.1)
PSA first, *n* (%)	207	188	395 (36.7)
PSA only	71	73	144 (13.4)
PSA > PAIN	57	50	107 (10.0)
PSA > PAIN > RADIO	28	23	51 (4.7)
PSA > RADIO	30	21	51 (4.7)
PSA > RADIO > PAIN	21	21	42 (3.9)
RADIO first, *n* (%)	62	72	134 (12.5)
RADIO only	28	30	58 (5.4)
RADIO > PAIN	22	25	47 (4.4)
RADIO > PAIN >PSA	5	7	12 (1.1)
RADIO > PSA	7	8	15 (1.4)
RADIO > PSA > PAIN	0	2	2 (0.2)
NO PROGRESSION, *n* (%)	59	63	122 (11.3)
PAIN w/o PSA rise *	131	152	283 (26.3)
RADIO w/o PSA rise ^+^	50	55	105 (9.8)

Each row name corresponds to a progression order. * is the sum of following patterns: PAIN only, PAIN > RADIO, RADIO > PAIN. ^+^ is the sum of following patterns: RADIO only, RADIO > PAIN. CABA20: cabazitaxel 20 mg/m^2^ every 3 weeks (Q3W), CABA25: cabazitaxel 25 mg/m^2^ Q3W. PAIN is defined as an increase of 1 point in the median PPI from its nadir noted on two consecutive three-week-apart visits or 25% increase in the mean analgesic score compared with the baseline score and noted on two consecutive three-week-apart visits. PSA is defined as follows: in PSA non-responders, progression is defined as an increase by at least 25% over the baseline value (at least 2 ng/mL) confirmed by a second value at least 3 weeks later; in PSA responders, progression is defined as a ≥25% increase over the nadir (at least 2 ng/mL), confirmed by a second value at least 3 weeks later. RADIO is defined as a radiological progression as per RECIST or PCWG2 criteria.

## Data Availability

Qualified researchers may request access to patient-level data and related documents [including, e.g. the clinical study report, study protocol with any amendments, blank case report form, statistical analysis plan, and dataset specifications]. Patient data will be anonymized, and study documents will be redacted to protect the privacy of trial participants. Further details on Sanofi’s data sharing criteria, eligible studies, and process for requesting access can be found at https://www.clinicalstudydatarequest.com (accessed on 8 February 2021).
